# Presynaptic Enhancement of Transmission from Nociceptors Expressing Nav1.8 onto Lamina-I Spinothalamic Tract Neurons by Spared Nerve Injury in Mice

**DOI:** 10.1523/ENEURO.0087-24.2024

**Published:** 2024-09-10

**Authors:** Wei-Chen Hung (洪瑋辰), Chih-Cheng Chen (陳志成), Cheng-Tung Yen (嚴震東), Ming-Yuan Min (閔明源)

**Affiliations:** ^1^Department of Life Science, College of Life Science, National Taiwan University, Taipei 10617, Taiwan; ^2^Neurobiology and Cognitive Science Centre, National Taiwan University, Taipei 10617, Taiwan; ^3^Institute of Biomedical Sciences, Academia Sinica, Taipei 11529, Taiwan

**Keywords:** brain slices, chronic pain, optogenetics, spinal cord, synaptic plasticity

## Abstract

Alteration of synaptic function in the dorsal horn (DH) has been implicated as a cellular substrate for the development of neuropathic pain, but certain details remain unclear. In particular, the lack of information on the types of synapses that undergo functional changes hinders the understanding of disease pathogenesis from a synaptic plasticity perspective. Here, we addressed this issue by using optogenetic and retrograde tracing ex vivo to selectively stimulate first-order nociceptors expressing Nav1.8 (NRs^Nav1.8^) and record the responses of spinothalamic tract neurons in spinal lamina I (L1-STTNs). We found that spared nerve injury (SNI) increased excitatory postsynaptic currents (EPSCs) in L1-STTNs evoked by photostimulation of NRs^Nav1.8^ (referred to as Nav1.8-STTN EPSCs). This effect was accompanied by a significant change in the failure rate and paired-pulse ratio of synaptic transmission from NRs^Nav1.8^ to L1-STTN and in the frequency (not amplitude) of spontaneous EPSCs recorded in L1-STTNs. However, no change was observed in the ratio of AMPA to NMDA receptor–mediated components of Nav1.8-STTN EPSCs or in the amplitude of unitary EPSCs constituting Nav1.8-STTN EPSCs recorded with extracellular Ca^2+^ replaced by Sr^2+^. In addition, there was a small increase (approximately 10%) in the number of L1-STTNs showing immunoreactivity for phosphorylated extracellular signal-regulated kinases in mice after SNI compared with sham. Similarly, only a small percentage of L1-STTNs showed a lower action potential threshold after SNI. In conclusion, our results show that SNI induces presynaptic modulation at NR^Nav1.8^ (consisting of both peptidergic and nonpeptidergic nociceptors) synapses on L1-STTNs forming the lateral spinothalamic tract.

## Visual Abstract

**Figure:**
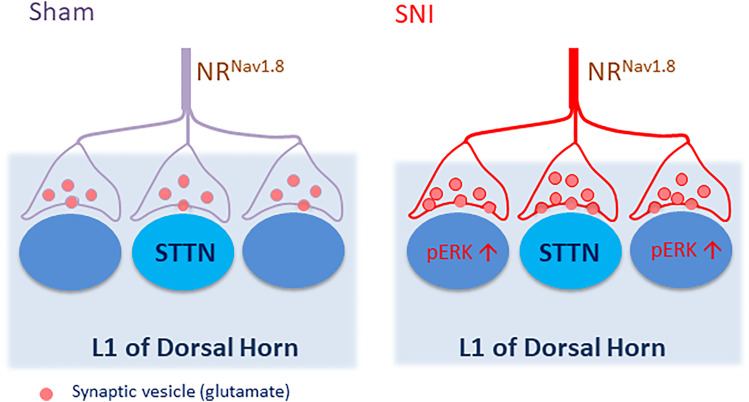
SNI results in increased glutamate release from nociceptors and no postsynaptic modulation in STTN, but other DH neurons show an increase in pERK levels.

## Significance Statement

Altered synaptic function in the dorsal horn (DH) has been suggested as a possible cellular substrate for neuropathic pain. However, the type of synapse involved remains unclear. This study uses optogenetics to activate Nav1.8-expressing nociceptors and retrograde fluorescence tracing for whole-cell recordings from spinothalamic tract neurons (STTNs). The results reveal a presynaptic enhancement of transmission at this synapse type following nerve injury. Given the diversity of synapse types in the spinal cord, these results provide a more specific insight into the cellular mechanisms of neuropathic pain by demonstrating increased efficacy of transmitter release from nociceptor axon terminals onto STTNs, which are the primary gatekeepers of the nociceptive pathway, transmitting noxious signals from the spinal cord to the primary somatosensory cortex.

## Introduction

Peripheral nerve injury caused by mechanical trauma, toxic chemicals, infection, and disease can lead to neuropathic pain—a chronic pain condition characterized by pain evoked in the absence of external stimulation (spontaneous pain), by innocuous stimulation (allodynia), or by increased pain responses to noxious stimulation (hyperalgesia; for review, see Costigan et al., 2009). Because of its profound impact on patients’ quality of life, the mechanism underlying the pathogenesis of neuropathic pain has received considerable attention (Costigan et al., 2009; O. Chen et al., 2020). Current evidence suggests that injured nerves not only become hypersensitive per se, with increased spontaneous activity generated both at the site of injury (neuroma) and at cell bodies in the dorsal root ganglia (DRG), but also exhibit increased spontaneous activity in their neighboring intact nerves (Blumberg and Janig, 1984; Wu et al., 2002; Sukhotinsky et al., 2004; Amir et al., 2005; Tseng et al., 2012). In addition, there are changes in the organization and strength of synaptic transmission from the central terminals of injured DRG neurons to their postsynaptic targets in the spinal dorsal horn (DH), leading to central sensitization (Woolf et al., 1992; H. Ikeda et al., 2003, 2006; Costigan et al., 2009). Central sensitization occurs not only in the DH but also at the synapses of nociceptive inputs to projection neurons in many supraspinal brain regions (the so-called pain matrix), such as the paraventricular nucleus of the thalamus, the central amygdala, and the anterior cingulate cortex in experimental animals with chronic pain (Wei and Zhuo, 2001; Neugebauer et al., 2003; R. Ikeda et al., 2007; W. K. Chen et al., 2010; Cheng et al., 2011; Min et al., 2011; Zhang et al., 2022).

Accumulating evidence has shown that the development of central sensitization in DH and the brain pain matrix share common molecular and cellular mechanisms with the induction and expression of long-term potentiation (LTP) of synaptic transmission in many cortical areas and the hippocampus (for review, see Ji et al., 2003; Latremoliere and Woolf, 2009). Although much has been learned in recent decades, some details remain to be elucidated. For example, the DH and the brain pain matrix contain a variety of afferent inputs and neuron types (Zholudeva et al., 2021), and it is unknown which synapse types undergo functional plasticity in neuropathic pain conditions. The lack of such information has limited our understanding of the mechanisms underlying neuropathic pain from the perspective of synaptic plasticity. In recent years, advances in optogenetics have made it possible to target a specific synapse type for investigation; therefore, synaptic plasticity in chronic pain and neuropathic conditions needs to be re-examined in a synapse-type–specific manner in the DH and brain pain matrix.

In this study, we re-examined whether there are functional changes in synaptic transmission from Nav1.8-expressing nociceptors (NR^Nav1.8^) in the DRG to spinothalamic tract neurons (STTNs) in spinal lamina I (L1), also known as the border zone of the DH, following spinal nerve injury (SNI), an animal model of persistent peripheral neuropathic pain (Decosterd and Woolf, 2000). This type of synapse (hereafter referred to as NR^Nav1.8^-STTN synapses) was targeted because it is the first gate of the nociceptive pathway that transmits noxious signals through the lateral spinothalamic pathway to the primary somatosensory (S-1) cortex (Willis, 1985) and is thought to be primarily responsible for sharp and localized pain occurring near the body surface (Kenshalo et al., 1980; Willis, 1985). The L1-STTNs receive monosynaptic contacts from axonal terminals of first-order nociceptive neurons in the DRG and have direct axonal projections to the ventral posterolateral nucleus of the thalamus (VPL). The axons decussate and join the contralateral anterolateral quadrant of the white matter, also known as the ascending lateral spinothalamic tract, along which most L1-STTN axons terminate in the VPL, which then relay nociceptive signals to the S-1 cortex. Using combined optogenetics for selective activation of NRs^Nav1.8^ (Stirling et al., 2005; Daou et al., 2016) and retrograde fluorescence tracing for whole-cell recording from the L1-STTNs, we report here a presynaptic but minor postsynaptic enhancement of transmission at NR^Nav1.8^-STTN synapses after SNI in mice.

## Materials and Methods

### Animals

Male offspring of Ai32 mice (strain #:012569, Jackson Laboratory) crossed with Nav1.8Cre mice (Stirling et al., 2005), referred to as Nav1.8-channelrhodopsin 2 (Nav1.8Chr2) mice, were used for all animal experiments. This choice of animal sex is intended to avoid the potential effects of estrous cycle-related hormones at this stage. However, as a growing number of studies in the field of pain indicate sex differences in pain, this issue should be systematically investigated as a separate study in the future. All experimental procedures were approved by the Institutional Animal Care and Use Committees, and every effort was made to minimize the number of mice used and their suffering. Mice were housed and maintained in a temperature-controlled vivarium under a 12 h light/dark cycle, with food and water available *ad libitum*.

### Surgery for stereotactic infusion of CTB and SNI and behavioral testing

Mice were deeply anesthetized, as indicated by the absence of the hindpaw withdrawal reflex, with an intraperitoneal (IP) injection of a mixture of ketamine (75 mg/kg) and xylazine hydrochloride (15 mg/kg), and additional aesthetic doses were administered as required. The mice were then mounted in a stereotaxic apparatus, small craniotomies were made in the skull, and the dura was reflected. A glass pipette with a tip diameter of 30–50 μm was loaded with 0.5% cholera toxin subunit B conjugated to CF594 (CTB-594; Biotium, cat #00072) and connected at the back to a 5.0 µl Hamilton syringe (Model 75 RN syringe, NDL sold separately, Hamilton). The glass pipette was then slowly advanced to the right ventrobasal complex of the thalamus (VB thalamus), and CTB-594 was unilaterally infused at three sites in the nucleus using the following coordinates (in mm) from the bregma: (1) AP −1.5, ML 1.5, DV 3.7; (2) AP −1.8, ML 1.5, DV 3.7; and (3) AP −2.1, ML 1.5, DV 3.2. For each infusion, 100 nl of CTB-594 was manually administered at a rate of 20 nl/min. In another series of experiments, in addition to CTB-594 infused into the VB, a single infusion of CTB-488 (Biotium, catalog #00070) was simultaneously made into the right lateral parabrachial nucleus (LPB) using the following coordinates (in mm) from the bregma: AP −5.34, ML 1.25 and DV 2.5. Due to the significantly smaller size of the LPB, the infusion volume was reduced to 30 nl to avoid diffusion of the tracer into the surrounding areas.

To measure behavioral responsiveness to mechanical stimulation, a period of 3 d was allowed for habituation to the test environment prior to the behavioral test. Two measurements were performed, one immediately before surgery for SNI and the other immediately before the preparation of spinal cord slices for electrophysiological recording. During the behavioral test, the mice were placed in an elevated acrylic box with a wire mesh floor. The plantar surface of the paw was stimulated with a series of ascending force von Frey filaments (in g): 0.008, 0.02, 0.04, 0.07, 0.16, 0.4, 0.6, and 1.0 (Touch Sensory Evaluators, North Coast Medical). Testing began with the 0.16 g filament. If a withdrawal response was observed, the lighter filament was used; if no response was observed, the heavier filament was used. The 50% withdrawal threshold was then determined using the up–down method (Yeh et al., 2021). Each procedure was repeated three times 10–15 min apart.

For SNI and sham surgery, anesthesia was induced as described above for stereotactic surgery. A skin incision was made on the lateral surface of the left thigh (contralateral to the CTB-594 infusion), followed by an incision directly through the biceps femoris to expose the sciatic nerve and its three terminal branches: sural, common peroneal, and tibial nerves. Axotomy was performed on the tibial and common peroneal nerves, leaving the sural nerve intact. Care was taken to avoid any contact or stretching of the intact sural nerve, and the muscle and skin were closed in two layers. In the sham control, the sciatic nerve and its branches were exposed without any lesions.

### Spinal cord slice preparation and electrophysiology

Mice were deeply anesthetized by intraperitoneal injection of urethane (1.5 g/kg) and perfused through their cardiovascular system with 10 ml of ice-cold slicing artificial cerebrospinal fluid (aCSF) consisting of the following (in mM): 92 *N*-methyl-d-glucose (NMDG), 2.5 KCl, 1.2 NaH_2_PO_4_, 10 MgSO_4_, 0.5 CaCl_2_, 20 HEPES, 30 NaHCO_3_, 25 glucose, 5 Na-ascorbate, and 3 Na-pyruvate, oxygenated with 95% O_2_ and 5% CO_2_, pH 7.3–7.4 and osmolality adjusted to 315–318 mOsm with sucrose. Dissection of the spinal cord for slicing followed the procedure described by Krotov et al. (2017). Briefly, the vertebral spine from thoracic 7 to sacral 1 segments (with ribs attached) was dissected after perfusion and transferred to a glass Petri dish filled with ice-cold slicing aCSF under a stereomicroscope (SZ61, Olympus). The spinal cord was exposed ventral side up after the pedicle was removed bilaterally using Vannas scissors (H-4240, Albert Heiss). The dura mater was then carefully cut above the ventral midline, and the spinal cord was removed from the lumbosacral enlargement.

The spinal cord was blocked from the lumbar 3–5 segments. In addition, the block was embedded in 3.5% UltraPure low melting point agarose (catalog #16520-050, Thermo Fisher Scientific) in slicing aCSF. Coronal spinal cord slices of 350 μm thickness were cut using a vibratome (Leica VT1000 S, Leica Biosystems), followed by incubation with slicing aCSF at room temperature (RT, 24–26°C) for 30 min. The slices were then incubated with holding aCSF containing the same ingredients as slicing aCSF, except that (in mM) 92 NaCl, 2 MgSO_4_, and 2 CaCl_2_ were substituted for 92 NMDG, 10 MgSO_4_ and 0.5 CaCl_2_, respectively. The slices were then transferred to a recording chamber mounted on an upright microscope equipped with Nomarski and epifluorescence optics (BX51WI, Olympus Optical) and an ORCA-R2 camera (Hamamatsu Photonics). The slices were continuously perfused with regular aCSF containing the following (in mM): 119 NaCl, 2.5 KCl, 1 NaH_2_PO_4_, 1.3 MgSO_4_, 2.5 CaCl_2_, 26.2 NaHCO_3_, and 11 glucose, oxygenated with 95% O_2_ and 5% CO_2_, pH 7.3–7.4 and osmolality 305–310 mOsm. To record L1-STTNs, CTB-594-labeled neurons in the left dorsal horn (DH) were selected for recording. Recordings were made under visual guidance using a glass pipette pulled from a borosilicate glass capillary (GC150F-10, Warner Instruments) with a tip resistance of 10–15 MΩ when filled with pipette solutions consisting of the following (in mM): 131 K-gluconate, 2 KCl, 10 HEPES, 2 EGTA, 8 NaCl, 2 ATP, and 0.3 GTP, pH 7.2–7.3 and osmolality 305 mOsm. For the AMPA/NMDA ratio experiment, K-gluconate was replaced by Cs-methanesulfonate, and KCl was replaced by CsCl. In a series of experiments, 10 mM biocytin was added to the pipette solution to fill the recorded neurons for morphological examination. Data were accepted only if the membrane potential (Vm) of the recorded cell was at least −50 mV and the action potentials (AP) were able to overshoot 0 mV. For voltage (*V*) clamp recordings, Vm was clamped at −70 mV unless otherwise stated. Serial resistance was monitored continuously throughout the recording, and data were excluded if serial resistance varied by >20% of the original value, which was typically <20 MΩ. All recordings were made at RT using a MultiClamp 700B amplifier (Molecular Devices), and the signals were low-pass filtered at 2 kHz. The signal was digitized at 10 kHz using a Micro1401 interface running Signal and Spike2 (Cambridge Electronic Design). In general, four slices were obtained from each mouse, and in most cases, data were collected from only one of the slices. We recorded only one L1-STTN per slice.

In optogenetic experiments, photostimulation was performed using 470 nm light pulses (duration, 2 ms, unless otherwise stated) generated by an LED source (LED4D067 and DC4100 Drivers, Thorlabs) and delivered through the epifluorescence light path and objective (40× water immersion lens, LUMPLFLN 40XW, Olympus) of the microscope. The LED light source was controlled by a Micro1401 MKII (Cambridge Electronic Design) to deliver a single or paired light pulse every 30 s. The LED light intensity was measured with a digital power meter (PM100D and s120c, Thorlabs) and adjusted in the range of 0.6–2 mW/cm^2^. All electrophysiological data were measured and analyzed using Signal and Spike2 (Cambridge Electronic Design).

### Histochemistry

To validate the CTB infusion pattern, the brain was rapidly dissected and blocked, and the brain block containing the thalamus and/or midbrain was kept in ice-cold slicing aCSF. After preparation of the spinal cord slices, the brain block was placed in 4% paraformaldehyde in 0.1 M phosphate buffer (PB) at 4°C overnight for fixation. After several brief rinses with PB, the brain block was soaked in 30% sucrose in PB for cryoprotection, followed by frozen sectioning. Tissue sections comprising the VB thalamus and LPB were mounted on slides using VECTASHIELD HardSet antifade mounting medium with DAPI (H-1500-10, Vector Laboratories) and examined using a Zeiss LSM 780 confocal microscope system (Carl Zeiss).

For post hoc examination of neuronal morphology, slices were fixed with 4% paraformaldehyde immediately after electrophysiological recording. Slices were subjected to previously described procedures for biocytin histochemistry without further sectioning. Briefly, after fixation, slices were incubated for 1 h at RT in phosphate-buffered saline (PBS: 0.9% NaCl in 0.01 M PB) containing 0.03% Triton X-100 (PBST) and 2% bovine serum albumin (BSA), followed by 2 h at RT with PBST-based solution containing a 1/200 dilution of streptavidin-conjugated Alexa Fluor 647 or DyLight 405 (catalog # 016-600-084 and catalog #016-470-084, Jackson ImmunoResearch). Between incubation steps, the sections were washed vigorously with PBST for at least three 10 min washes, and all incubations were performed at RT (unless otherwise specified). Sections were then mounted on slides with RapiClear 1.47 and examined using the Leica TCS SP8 confocal microscope system (Leica Microsystems). Image stacks containing the structural elements of the biocytin-filled STTN were reconstructed using Amira software (Thermo Fisher Scientific).

### Visualization of CTB-traced STTN and immunohistochemistry (IHC) for pERK

After CTB injection, mice were deeply anesthetized with urethane (1.5 g/kg) and then perfused via the cardiovascular system with 4% paraformaldehyde in PB. The spinal cord was dissected and blocked from L2 to L5. The spinal cord block was postfixed overnight at 4°C with the same fixative. The spinal cord block was then soaked in 30% sucrose in 0.1 M PB at 4°C for 24 h for cryoprotection. Using a freezing microtome, the spinal cord block was cut into 100-μm-thick sections after sinking in the sucrose solution. To block nonspecific reactions, the sections were rinsed briefly with PB, followed by PBS, and then incubated with 2% BSA in PBST for 1 h. In the series of experiments designed to evaluate L1-STTN showing immunoreactivity (IR) to an antibody against phosphorylated extracellular signal-regulated kinase (pERK) after SNI surgery, the sections were incubated overnight at 4°C in rabbit anti-pERK (dilution, 1:400; catalog #4370, Cell Signaling Technology) followed by incubation for 2 h in a 1/200 dilution of donkey anti-rabbit IgG conjugated to Alexa Fluor 488 (catalog #711-545-152, Jackson ImmunoResearch). For the procedures described above, sections were washed with PBST for at least three 10 min washes between incubation steps. All incubations were performed at RT (unless otherwise stated). For the series of experiments designed to detect L1-STTN projecting to the VB and/or LPB, sections were examined directly without further processing. In all cases, sections were mounted on slides with RapiClear 1.47 and examined with a Zeiss LSM 780 confocal microscope system.

In the series of experiments designed to evaluate L1-STTN exhibiting pERK-IR after SNI surgery, neurons located in the L1 of the DH exhibiting CTB-CF594 signal, pERK-IR, and both signals were counted for each section. Similarly, for the series of experiments designed to detect L1-STTN projecting to the VB and/or LPB, neurons located in the L1 of the DH exhibiting CTB-CF594 signal, CTB-CF488, and both signals were counted for each section. The brightness and contrast of the images were modified, and the background noise was then subtracted using ImageJ software (National Institutes of Health). To determine the background noise, three regions of interest (ROIs) that did not cover the DH were defined, the mean and SD of the mean pixel intensity in the three ROIs were calculated, and the background noise was taken as the mean plus twice the standard deviation. After subtraction, the aggregation of the remaining pixels into a recognizable soma structure was accepted as a neuron that was pERK-IR, CTB-CF594, and/or CTB-488 positive.

### Statistical analysis

For statistical comparisons, the normality of the data was first tested using the Shapiro–Wilk test. The Student's *t* test, paired *t* test, one-way ANOVA, or two-way ANOVA was used to compare two groups that both showed normal distributions; otherwise, the nonparametric paired Wilcoxon signed-rank test or Mann–Whitney *U* test was used. Paired *t* test and nonparametric paired Wilcoxon signed-rank test were used to compare data collected from a single neuron or mouse before and after drug application; Student's *t* test and Mann–Whitney *U* test were used for two independent groups. One-way ANOVA was used for comparisons between three or more groups. Two-way ANOVA was used for experiments with more than two variables, and post hoc analyses were performed using Tukey's multiple-comparisons test. All statistical analyses were performed using OriginPro 8 (OriginLab) and Microsoft Excel (Microsoft). All data are presented as mean ± SEM (standard error of the mean).

## Results

### Identification of L1-STTNs for whole-cell recording: the morphology study

**Figure 1.**
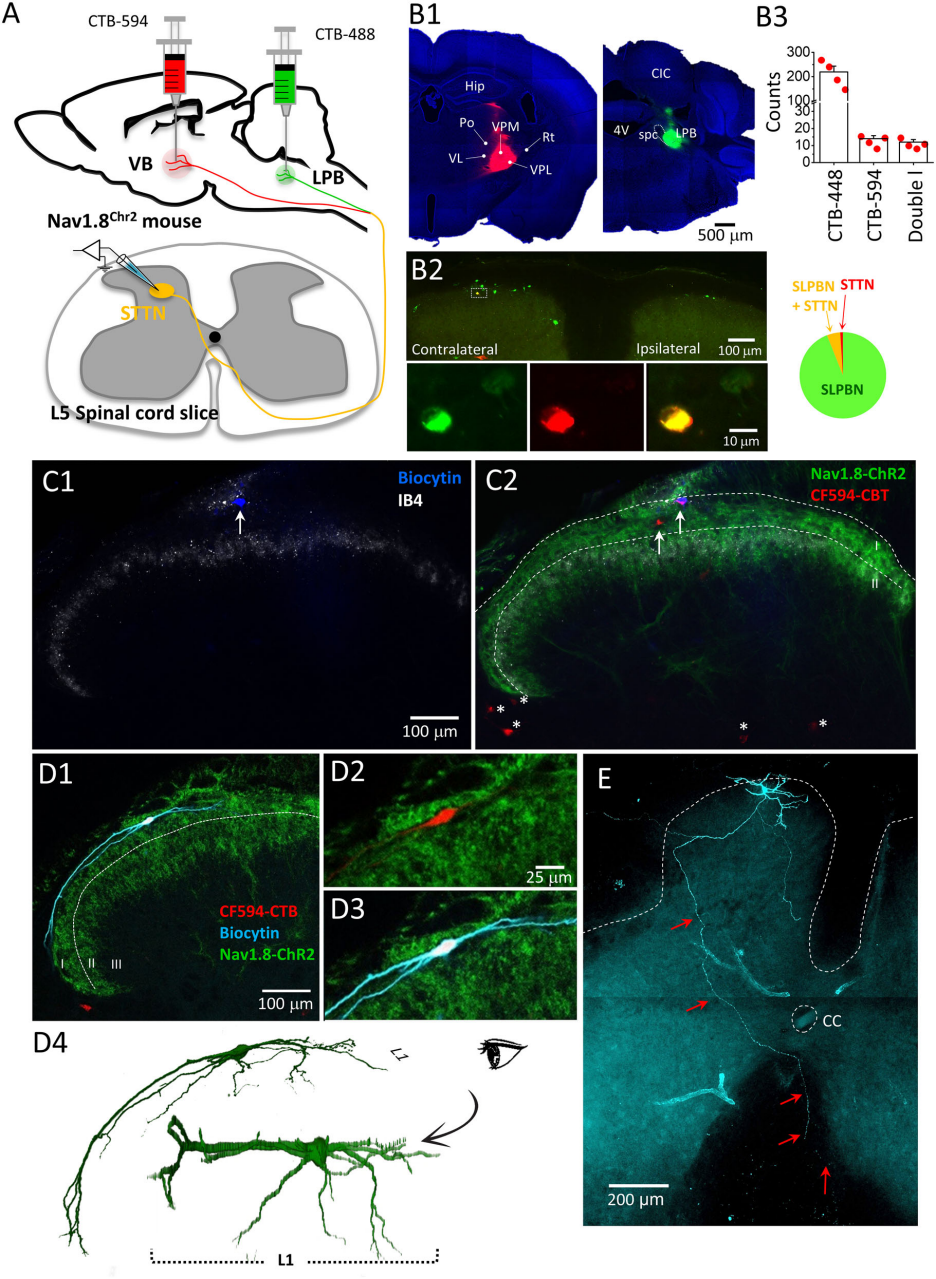
Identification of L1-STTNs for whole-cell recording and their morphology. ***A***, Schematic diagram showing the experimental setup for simultaneous labeling of STTNs and SLPBNs in spinal cord lamina I. ***B***, Images from a representative experiment demonstrate that CTB-594 and CTB-488 were deposited precisely in the VB (left panel, ***B*1**) and LPB (right panel, ***B*1**), resulting in labeling of some neurons with CTB-488 and few neurons with CTB-594 (***B*2**). Enlargement of the area enclosed by the dashed line (***B*2**) shows a DH neuron taking up the deposited CTB-488 (left panel, ***B*3**) and CTB-594 (middle panel, ***B*3**) as evidenced by the overlay of the two fluorophores (right panel, ***B*3**). Pooled results from four experiments (top histogram, ***B*3**) show the total number of neurons that take up CTB-488 (LPB-projecting neurons), CTB-594 (VB-projecting neurons), and the two fluorophores (LPB- and VB-projecting neurons) in the lumbar segments. Each circle represents a single experiment; the bar and the capped line represent the mean counts and the standard error of the mean, respectively. The bottom pie chart summarizes the percentage of DH neurons projecting to the LPB (SLPBN), VB (STTN), and LPB and VB (SLPBN + STTN). 4V, fourth ventricle; CIC, central nucleus of the inferior colliculus; LPB, lateral parabrachial nucleus; scp, superior cerebral peduncle; Po, posterior thalamus; Rt, reticular thalamic nucleus; VL, ventrolateral thalamus; VPL, ventral posterolateral thalamus; VPM, ventral posteromedial thalamus. ***C***, A representative experiment designed to label L1-STTNs for whole-cell recording. Images show a post hoc examination of a spinal cord slice cut from a mouse that received CTB-594 infusion into the VB. The slice was subjected to histochemistry for biocytin (***C*1**, blue) and CTB-594 (***C*2**, red) and IHC for IB4 (***C*1**, white) and eYFP-Chr2 (***C*2**, green). Note the two CTB-594 labeled neurons (marked with arrows) and the right cell is labeled and filled with biocytin. Note also the three CTB-594-labeled neurons in the lateral spinal nucleus and two in the deep lamina layers (marked with asterisks). ***D***, Another representative experiment shows the reconstruction of L1-STTN morphology after electrophysiological recording (***D*1**). The neuron is labeled with CTB-594 (***D*2**) and filled with biocytin (***D*3**), and its morphology is reconstructed (***D*4**). The image inserted at the bottom of the reconstruction shows a 90° rotation along the lateromedial axis to better illustrate that dendritic arborization is restricted within L1. ***E***, Another representative experiment shows the post hoc examination of the morphology of an L1-STTN (labeled with CTB-594 and filled with biocytin) after electrophysiological recording. Note the axon of the L1-STTN (marked with arrows) on its way to the contralateral side.

To identify DH neurons that reside in L1 and project to the thalamus, we infused CTB-594 into the right VB thalamus, which includes the VPL and the ventral posteromedial nucleus of the thalamus, in Nav1.8^Chr2^ mice. Since L1 neurons projecting to the LPB are thought to represent the majority of projection neurons in L1 (Spike et al., 2003; Wercberger and Basbaum, 2019; Choi et al., 2020), we simultaneously infused CTB-488 into the right LPB for comparison in this series of experiments (Fig. 1*A*). We estimated that the number of labeled L1 neurons projecting to the LPB collected from lumbar segments 2–5 contralateral to the CTB-594 injection site was 220.3 ± 24.4 (*n* = 4 mice), far exceeding the number (13.8 ± 1.8 cells) of VB projections collected from the same spinal segments (Fig. 1*B*). This finding is consistent with a previous study (Al-Khater et al., 2008) that reported a small number of L1 projection neurons to the thalamus. Impressively, up to 88.4 ± 5.5% of L1 neurons projecting to the VB also projected to the LPB, consistent with previous findings (Spike et al., 2003; Wercberger and Basbaum, 2019). This observation suggests that most L1 neurons that project to the VB are also projection neurons to the LPB. From another perspective, it suggests that only a very small fraction (∼6%) of L1 projection neurons to the LPB also project to the VB thalamus. Because previous studies have reported plastic changes in the strength of synaptic input to L1 neurons projecting to the LPB in inflammatory pain (H. Ikeda et al., 2003, 2006), we focused on L1 neurons projecting to the VB of the thalamus in this study.

To record L1 neurons projecting to the VB in whole-cell configuration, we prepared ex vivo spinal cord slices from the contralateral lumbar 3–5 segments of Nav1.8^Chr2^ mice that had received an infusion of CTB-594 into the right VB 7–10 d earlier (Fig. 1*A*). This mouse line is the offspring of Nav1.8^Cre^ mice with Cre recombinase expression driven by the promoter of the SCN10A gene, crossed with Ai32 mice carrying a knock-in gene expressing a fusion protein consisting of an enhanced channelrhodopsin 2 (Chr2) and an enhanced yellow fluorescent protein (eYFP) in a Cre-dependent manner. The SCN10A gene encodes a subunit of the voltage-gated sodium channel (Nav1.8) and is activated in a subset of DRG neurons, >85% of which are nociceptors (Akopian et al., 1996; Djouhri et al., 2003), hereafter referred to as NR^Nav1.8^; accordingly, Nav1.8^Chr2^ mice have a large number of NR^Nav1.8^-expressing Chr2-eYFP for optogenetic stimulation. Thus, the experimental setup described above allows not only selective labeling of L1 neurons projecting to the VB for whole-cell recording but also selective activation of NR^Nav1.8^. Furthermore, Prabhakar et al. (2019) reported a very limited level of leaky “off-target” ChR2-eYFP expression throughout the nervous system in Nav1.8^Chr2^ mice.

In the DH of spinal cord slices prepared from Nav1.8^Chr2^ mice receiving CTB-594 infusion into the right VB, there was a very dense Chr2-eYFP signal in L1 and spinal lamina II (L2), identified by immunohistochemistry (IHC) with the IB4 antibody (Fig. 1*C*; Snider and McMahon, 1998). This feature indicates that the central terminals of NR^Nav1.8^ are predominantly located in the superficial DH layers (L1 and L2), which is consistent with the observation made in a previous study using a similar approach (Daou et al., 2016) and the previous notion that the majority of DRG neurons expressing Nav1.8 are nociceptors (Akopian et al., 1996; Djouhri et al., 2003). Consistent with the observation made in the last series of experiments, the number of neurons labeled by retrograde CTB-594 in L1 was low, with generally approximately two cells identified per slice, although the number was slightly higher (up to four cells) in the deeper layers (Fig. 1*C*). Furthermore, the CTB-594–labeled neurons in L1 were tightly surrounded by the Chr2-eYFP signal (Fig. 1*C,D*). We targeted the CTB-594–labeled neurons in L1 for whole-cell recording and simultaneously filled them with biocytin. The results showed that most of the recorded neurons had 2–4 main dendrites spanning the entire mediolateral dimension of the DH, with the arborizations mostly restricted to L1 (Fig. 1*D*). In some cases, axons of the biocytin-filled neurons could be traced across the midline to the contralateral side (Fig. 1*E*), consistent with the argument that the recorded neurons were projection neurons. Accordingly, we considered them to be projection neurons to the VB in L1 (hereafter referred to as L1-STTNs), and all L1-STTNs described below were labeled with CTB-594 as described above.

### Recording of Nav1.8-STTN excitatory postsynaptic currents (EPSCs)

**Figure 2.**
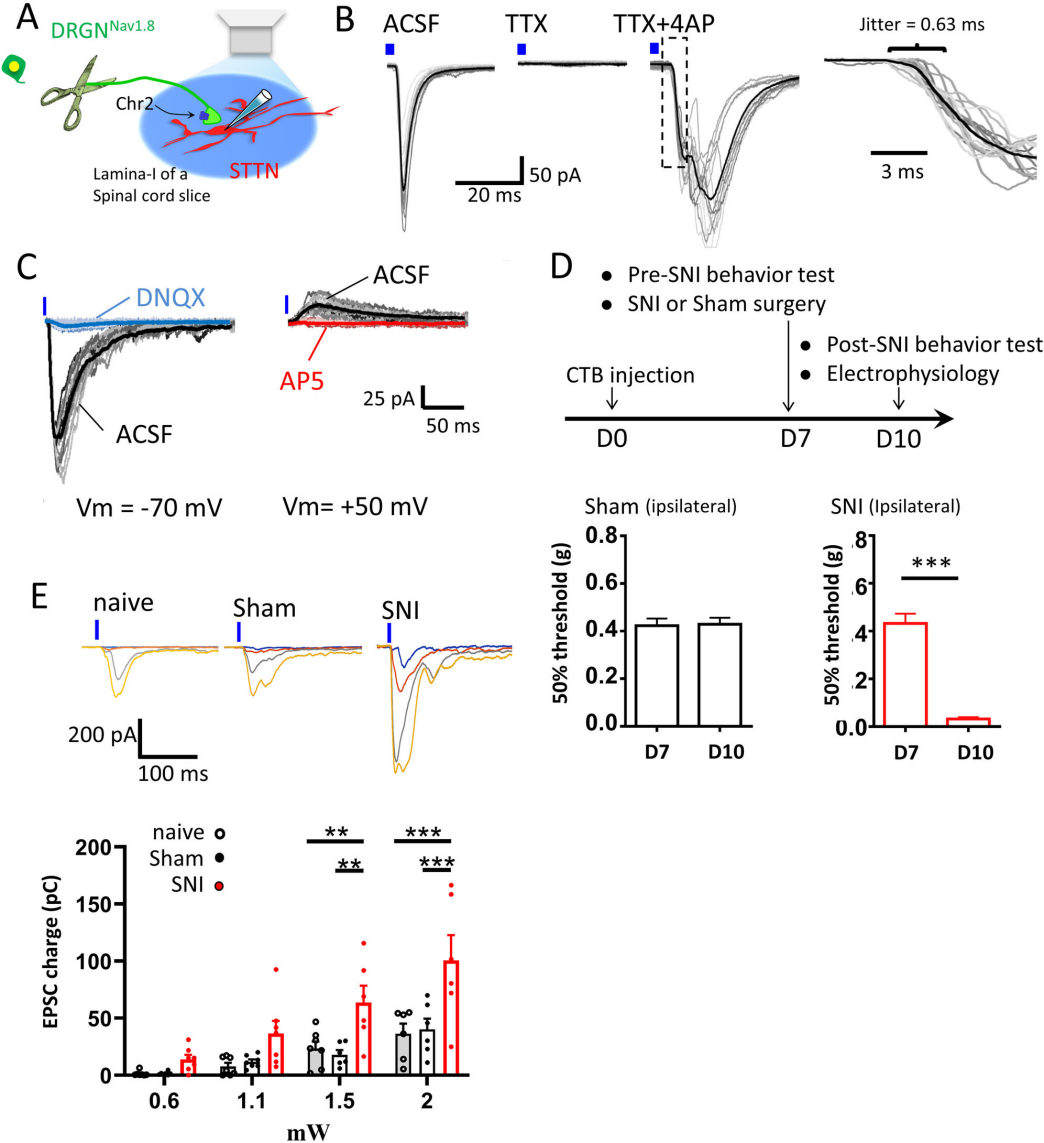
Recording of Nav1.8-STTN EPSCs and enhancement of the activity after SNI. ***A***, Schematic of the optogenetic setup for recording Nav1.8-STTN EPSCs. ***B***, A representative recording shows photostimulation (blue bar) evoked inward currents in an L1-STTN (*Vm* = −70 mV) during baseline (left), addition of TTX (middle), and addition of TTX + 4-AP (right). Superimposed light gray traces show sequential individual responses and the black trace shows the average response to light stimulation. The inset plot on the right is part of an enlargement of the recording in TTX + 4-AP, as indicated by the dashed rectangle. It shows the variation of the EPSC onset latency with a jitter of 0.63 ms. ***C***, A representative recording shows that Nav1.8-STTN EPSCs consist of AMPAR- (left) and NMDAR-mediated (right) components. Superimposed light gray, cyan, and red traces show sequential individual responses, and the black, cyan, and red traces show the averaged response to light stimulation. ***D***, The top timeline illustrates the experimental setup for tracer injection, SNI or sham surgery, behavioral testing, and electrophysiological recording (top). Bottom histograms show behavioral responses to mechanical stimuli with von Frey hairs from sham (left panel) and SNI (right panel) mice. The asterisk indicates a significant difference at the *p* < 0.001 level. ***E***, Raw data traces on top show representative recordings of Nav1.8-STTN EPSCs evoked by increasing light intensities in slices from naive (left), sham (middle), and SNI (right) mice, with each overlayed trace representing the averaged response of at least 10 sequential sweeps to light stimulation at a stimulating intensity. The plot at the bottom shows the pooled results. Each symbol in the plot indicates a single experiment, and the bars and dashed lines indicate the mean and standard error of the mean, respectively. The asterisks indicate significant differences at *p* < 0.01 (**) and <0.001 (***).

Photostimulation of the slices through the objective lens with a pulse of blue light-evoked inward synaptic currents in the L1-STTNs with Vm clamped at −70 mV (Fig. 2*A,B*). To isolate monosynaptic activity, we adopted the commonly used protocol for optogenetic evocation of monosynaptic currents (Petreanu et al., 2009), in which 1 μM tetrodotoxin (TTX) was first applied to block all action potential (AP)–dependent activity, followed by 100 μM 4-AP, a broad-spectrum voltage-gated K^+^ channel blocker, to enhance membrane depolarization of photosensitive axonal terminals, thereby promoting neurotransmitter release and restoring monosynaptic activity. We found that monosynaptic currents could be successfully evoked in the L1-STTN by photostimulation of NR^Nav1.8^ under these conditions (Fig. 2*B*), and the results were reproducible in six L1-STTNs from five mice. We also analyzed the latency of the evoked synaptic currents by measuring the time difference between the onset of the light pulse and the synaptic currents (Fig. 2*B*, inset to the right). The mean latency averaged over the six L1-STTNs (five mice) was 8.21 ± 0.71 ms with a mean latency jitter, defined as the mean of the standard deviation of the latency of a single evoked synaptic current, of 1.1 ± 0.2 ms. This result meets the criteria for monosynaptic activity reported in a previous study that also used optogenetics to characterize synaptic transmission from nociceptors expressing Mas-related G-protein–coupled receptor D to DH neurons in L2 (Wang and Zylka, 2009). Therefore, despite the known GABA-mediated presynaptic inhibition of nociceptor input to STTNs driven by the nociceptive afferent per se (Eccles et al., 1963; Fernandes et al., 2020), we chose not to use pharmacological agents to block GABA receptor functions for the electrophysiological recording described below. This decision was made because the use of optogenetics could allow specific NR^Nav1.8^ activation ex vivo. Namely, in contrast to conventional electrical stimulation experiments, this approach maintained the advantage of recording in a more physiological condition, without inhibitory synaptic transmission blockers to isolate excitatory synaptic activity. In addition, the following recordings of Nav1.8-STTN EPSCs were performed in the presence of TTX and 4-AP in the bathing medium to ensure the recording of monosynaptic activity, thereby minimizing the potential confounding effects of polysynaptic GABA-mediated activity.

In another series of experiments under the conditions described above, bath application of 10 μM DNQX, an AMPA receptor (AMPAR) antagonist, suppressed light-evoked synaptic currents. In addition, when Vm was subsequently clamped at +50 mV, the recorded L1-STTN responded to light stimulation with an outward current that was blocked by further application of 50 μM AP5, an NMDA receptor (NMDAR) antagonist (Fig. 2*C*). The same observations were repeated in 11 L1-STTNs (eight mice). Taken together, these results show that using ex vivo spinal cord slices cut from the lumbar 3–5 segments of Nav1.8^Chr2^ mice receiving CTB-594 infusion into the VB thalamus, we were able to selectively evoke EPSCs mediated by both AMPAR and NMDAR in the L1-STTN upon photostimulation of NR^Nav1.8^ axonal terminals. Herein, we refer to these EPSCs as Nav1.8-STTN EPSCs.

### SNI enhances Nav1.8-STTN synaptic transmission through presynaptic modulation

Since L1-STTNs are located at the first gate of the pain pathway to the brain, we investigated the effect of peripheral nerve injury on synaptic transmission of NR^Nav1.8^ to the L1-STTNs. To accomplish this (Fig. 2*D*), Nav1.8^Chr2^ mice received an infusion of CTB-594 into the right VB thalamus on the day of the experiments (defined as D0). On D7 (1 week later), SNI or sham surgery was performed on the left legs (contralateral to the CTB-CF594 infusion) after measuring the animals’ baseline behavioral responses to mechanical stimulation. On D10 (3 d after the surgery), we first measured an additional behavioral response to mechanical stimulation, and then the mice were killed for ex vivo spinal cord slice preparation and electrophysiological recording. We determined the time window for the electrophysiological study based on our preliminary results, which indicated a peak of behavioral hypersensitivity followed by stabilization on D3 after SNI. Therefore, the window for electrophysiological recording likely represents a transition period from pain chronification to pain maintenance.

Compared to all sham mice used for subsequent electrophysiological studies, which showed no significant change between D7 (0.43 ± 0.02 g) and D10 (0.44 ± 0.02 g, *n* = 49 mice; *p* = 0.82, paired *t* test), the SNI mice showed a significant reduction in the 50% threshold for the withdrawal response from 0.45 ± 0.04 g at D7 to 0.05 ± 0.00 g at D10 (Fig. 2*D*; *n* = 51 mice; *p* < 0.0001, paired *t* test). When we examined the input–output relationship of Nav1.8-STTN EPSCs, defined as the relationship between the gradually increasing light intensity of photostimulation and the corresponding EPSC charge transfer, we observed an increasing EPSC charge transfer with increasing light intensity at 0.6, 1.1, 1.5, and 2 mW/cm^2^ in recordings from naive (*n* = 7 L1-STTNs from five mice), sham (*n* = 7 L1-STTNs from six mice), and SNI (*n* = 7 L1-STTNs from five mice) slices (Fig. 2*E*; two-way AVOVA test, *p* < 0.0001; post hoc Tukey's multiple-comparisons test indicates a significant difference in EPSC charge transfer between 0.6 vs 2 at *p* = 0.0024 and 1.5 vs 2 at *p* = 0.0174 in the naive group; 0.6 vs 2 at *p* = 0.0009 and 1.5 vs 2 at *p* = 0.0208 in the sham group; and 0.6 vs 1.5 at *p* < 0.0001 and 0.6 vs 2 at *p* < 0.0001, 1.1 vs 1.5 at *p* = 0.0091, 1.1 vs 2 at *p* < 0.0001, and 1.5 vs 2 at *p* = 0.0021 in the SNI group). In addition, EPSC charge transfer recorded from SNI mice was larger than that from naive or sham mice at all light intensities, with the difference reaching significant levels at 1.5 and 2 mW/cm^2^; there was no difference between sham and naive mice (two-way ANOVA test, *p* < 0.0001; post hoc Tukey's multiple-comparisons test shows a significant difference between sham vs SNI at 1.5 and 2 mW/cm^2^ with *p* = 0.0024 and *p* < 0.0001, respectively, and naive vs SNI at 1.5 and 2 mW/cm^2^ with *p* = 0.0059 and *p* < 0.0001, respectively; Fig. 2*E*). These results indicate a light intensity-dependent increase in charge transfer of optogenetically evoked Nav1.8-STTN EPSCs and that SNI enhances the strength of the synaptic transmission.

**Figure 3.**
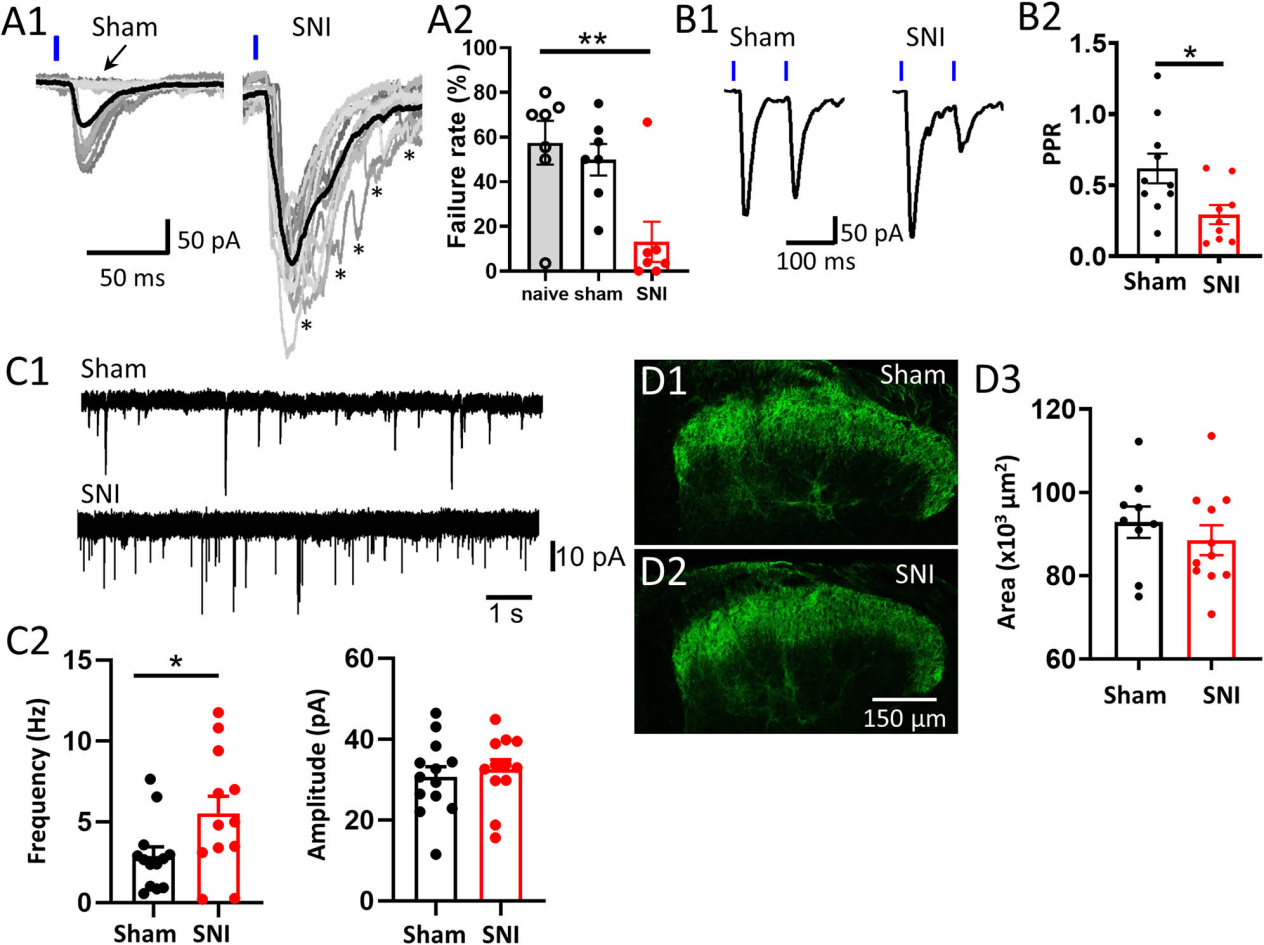
Enhancement of Nav1.8-STTN EPSCs by SNI involves presynaptic modulation. ***A***, Raw data traces show representative recordings of Nav1.8-STTN EPSCs evoked by light stimulation at an intensity of 1.1 mW/cm^2^ in slices from sham and SNI mice (***A*1**) and a plot showing the pooled results (***A*2**). Note the appearance of failure responses in the sham slice (marked with an arrow) but not in the SNI slice. Also note the asynchronous events (marked with asterisks) in the recording from the SNI slice but not from the sham slice. In the recording of Nav1.8-STTN EPSCs (***A*1**), each superimposed gray trace represents an individual response and the black trace represents the averaged response to light stimulation. ***B***, Raw data traces show representative recordings of the PPR of Nav1.8-STTN EPSCs in slices from sham and SNI mice (***B*1**), and a plot shows the pooled results (***B*2**). ***C***, Raw data traces show representative recordings of sEPSCs in L1-STTNs from sham and SNI mice (***C*1**), and a plot shows the pooled results of frequency (left, ***C*2**) and amplitude (right, ***C*2**) analyses. ***D***, Representative images show Chr2-eYFP expression in spinal cord slices from sham (***D*1**) and SNI (***D*2**) mice, and a plot shows the pooled results. For all plots shown (***A*2**, ***B*2**, ***C*2**, ***D*2**), each symbol (black or red circle) in the plots denotes the recording from an L1-STTN (***A*2**, ***B*2**, ***C*2**) or one slice (***D*2**); bars denote the means, and capped lines denote the standard errors of the means. The asterisks indicate significant differences at the *p* < 0.05 (*) or <0.01 (**).

Next, we investigated the mechanisms behind the increased strength of synaptic transmission after SNI. First, we examined the failure rate (FR) of Nav1.8-STTN EPSCs in response to photostimulation, using the same data to construct the input–output relationship described above. The results show that the enhancement of Nav1.8-STTN EPSCs by SNI was associated with a significant reduction in FR. The FR averaged over seven L1-STTNs from six SNI mice, seven L1-STTNs from five sham mice, and seven L1-STTNs from five naive mice was 13.1 ± 8.4%, 49.9 ± 6.5%, and 57.5 ± 9.1%, respectively (one-way ANOVA test: *p* = 0. 0045; post hoc Tukey's multiple-comparisons test indicates a significant difference between SNI and sham with *p* = 0.0207 and between SNI and naive with *p* = 0.0055, but not between sham and naive with *p* = 0.81; Fig. 3*A*). These results preliminarily suggest the involvement of presynaptic modulation in the effect of SNI. To strengthen this argument, we also examined the ratio of Nav1.8-STTN EPSC amplitude evoked by the second pulse to that evoked by the first pulse after delivery of a pair of light pulses (PPR) with an inert pulse interval of 100 ms. Because no significant difference was observed between sham and naive mice in the Nav1.8-STTN EPSC input–output relationship and in the FR, we collected data only from sham mice for comparison in this and the following series of studies. For the PPR, the results showed a significant difference between recordings from SNI and sham mice. The PPR averaged over 10 L1-STTNs from 10 SNI mice and 10 L1-STTNs from 10 sham mice was 0.31 ± 0.06 and 0.62 ± 0.10, respectively (*p* = 0.0209, unpaired *t* test; Fig. 3*B*).

In addition to FR and PPR, we analyzed spontaneous EPSCs (sEPSCs) recorded in the L1-STTN under regular conditions (no TTX and 4-AP added; Fig. 3*C*1) and observed a significantly higher frequency of sEPSCs in slices from SNI mice compared with sham mice. The mean frequency of sEPSCs, averaged over 12 L1-STTNs from 12 SNI mice and 13 L1-STTNs from 13 sham mice, was 5.50 ± 0.56 Hz and 2.85 ± 0.59 Hz, respectively (*p* = 0.0405, Mann–Whitney *U* test; Fig. 3*C*2 left). No difference in amplitude was found between the two groups; the mean amplitude, averaged over the 12 L1-STTNs from SNI mice and the 13 L1-STTNs from sham mice, was 32.50 ± 2.44 pA and 30.59 ± 2.58 pA, respectively (*p* = 0.60, unpaired *t* test; Fig. 3*C*2 right). These results are consistent with a previous study that reported a significant difference in the mean frequency, but not amplitude, of sEPSCs recorded in projection neurons expressing the NK-1 receptor in L1 after SNI (Y. Liu et al., 2017).

Finally, SNI did not significantly alter the Chr2-eYFP expression profile in the left DH (ipsilateral to SNI) of the spinal cord slices (Fig. 3*D*). Comparison of spinal cord slices from SNI and sham mice revealed no significant difference in the area of Chr2-eYFP signal in the left DH between the two groups (*n* = 11 slices from eight SNI mice and 9 slices from six sham mice, *p* = 0.74, Mann–Whitney *U* test). The mean Chr2-eYFP signal areas of the SNI slices and sham mice were 88.5 ± 3.6 × 10^3^ µm^2^ and 92.9 ± 3.8 × 10^3^ µm^2^, respectively. Taken together, these results from FR, PPR, and sEPSC analysis indicate a presynaptic enhancement of synaptic transmission from NR^Nav1.8^ to the L1-STTN after SNI. Moreover, SNI did not seem to induce severe degeneration or sprouting of the central terminals of injured and spared NR^Nav1.8^ axons. Meanwhile, there was no difference in mean sEPSC amplitude between recordings made in the L1-STTN of SNI and sham mice, suggesting no involvement of postsynaptic modulation in the enhancement of Nav1.8-STTN EPSCs after SNI.

### SNI-induced enhancement of Nav1.8-STTN EPSCs does not involve postsynaptic modulation

**Figure 4.**
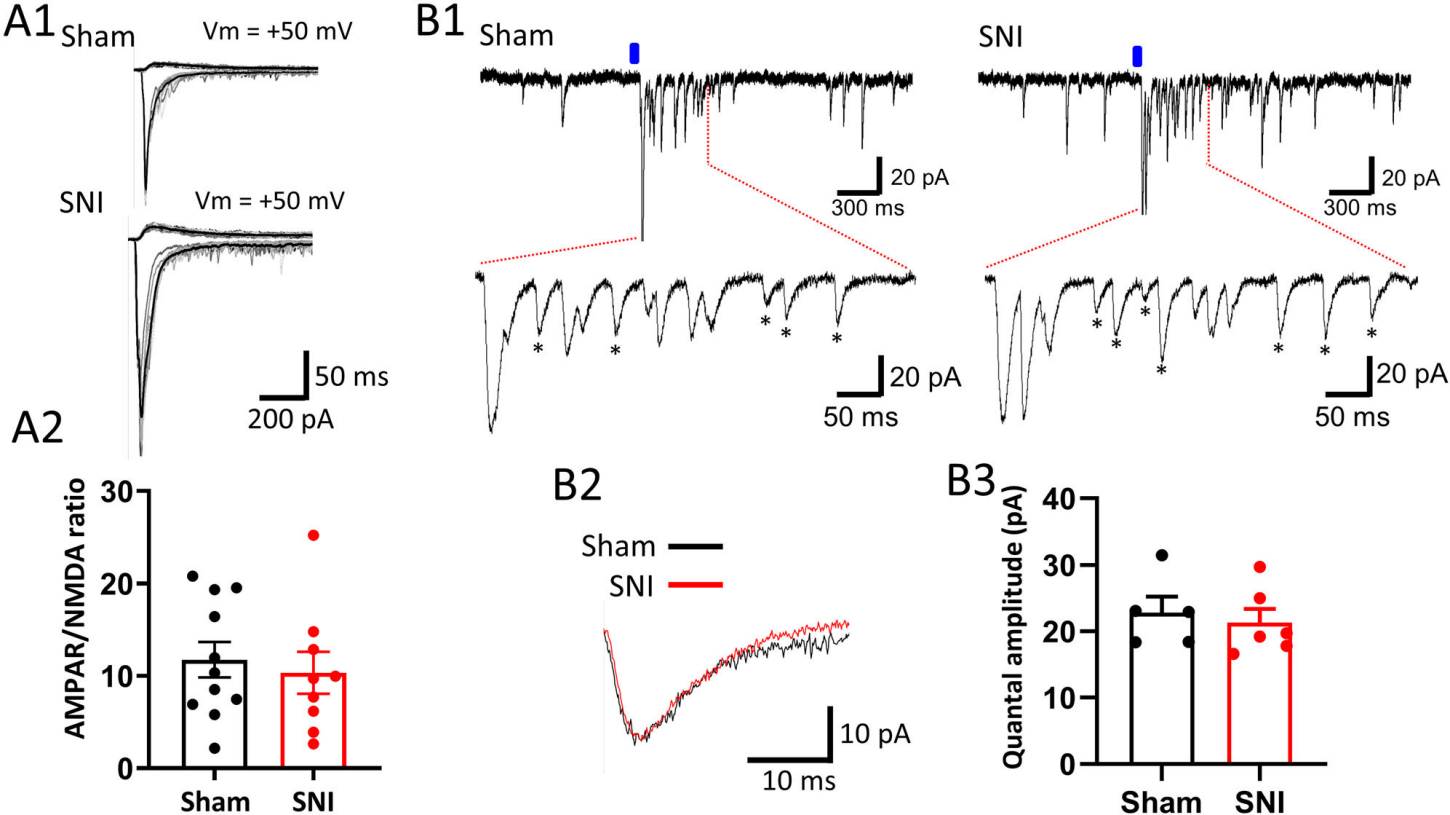
The enhancement of the Nav1.8-STTN EPSC transmission after SNI is not associated with a change in the AMPAR/NMDAR ratio nor with a change in the mean amplitude of the aEPSCs. ***A***, Raw traces show representative recordings of AMPAR- (top, ***A*1**) and NMDAR-mediated (bottom, ***A*1**) components of Nav1.8-STTN EPSCs from L1-STTNs of sham and SNI mice, and a plot shows the pooled results (***A*2**). Superimposed light gray traces show sequential individual responses and the black trace shows the average response to light stimulation. ***B***, Raw data traces show representative recordings of Nav1.8-STTN aEPSCs in 1 mM Sr^2+^ from sham (left, ***B*1**) and SNI (right, ***B*1**) mice. The asterisks mark the individual Nav1.8-STTN aEPSC events that were averaged to compare their mean amplitude between recordings in the sham and SNI slices (***B*2**). The lower right plot shows the pooled results (***B*3**). Each symbol in the plots (***A*2**, ***B*3**) denotes a recording from a mouse L1-STTN; bars denote the means, and capped lines denote the SEM.

To further support the argument of no postsynaptic modulation of the SNI effect, we analyzed the ratio of the AMPA receptor (AMPAR)–mediated component to the NMDA receptor (NMDAR)–mediated component of Nav1.8-STTN EPSCs and compared the data collected from SNI and sham mice. This parameter was analyzed because it is a widely accepted indicator of modulation of synaptic function involving postsynaptic modulation, based on the fact that the expression of various forms of synaptic plasticity, including long-term potentiation (LTP) in the hippocampus, is mediated by modulation of the number of postsynaptic ionotropic glutamate receptors, in particular the regulated cycling of specific AMPARs and their trafficking to the cell surface (Shi et al., 1999; Hayashi et al., 2000; Lee et al., 2000; Malinow and Malenka, 2002). To collect data on this parameter, we first recorded Nav1.8-STTN EPSCs with Vm clamped at −70 mV to sample the AMPAR-mediated component, followed by recording with the addition of 10 μM DNQX to the bath medium and Vm clamped at +50 mV to sample the NMDAR-mediated component (Fig. 4*A*1). The charge transfers of the two components were measured, and the ratio was taken. The AMPAR/NMDAR ratio of Nav1.8-STTN EPSCs showed no difference between recordings in slices from SNI and sham mice; the ratio averaged over 11 L1-STTNs from eight SNI mice and 11 L1-STTNs from eight sham mice was 10.3 ± 2.3 and 11.7 ± 1.9, respectively (*p* = 0.66, Mann–Whitney *U* test; Fig. 4*A*2).

Results from previous studies have shown that activation of NMDARs in the dorsal horn plays a critical role in the development of behavioral hypersensitivity following peripheral tissue damage or nerve injury (Woolf and Salter, 2000); furthermore, intrathecal administration of NMDAR antagonists (Seltzer et al., 1991; Yamamoto and Yaksh, 1992) or siRNA targeting the NR2B subunit (Tan et al., 2005) reduces nociceptive behavior and pain hypersensitivity induced by nerve injury. Accordingly, it is possible that there was a parallel increase in AMPAR and NMDAR functionality, countering the postsynaptic modulation argument and explaining the lack of significant change in the AMDAR/NMDAR ratio of Nav1.8-STTN EPSCs after SNI. To test this possibility, we directly compared the amplitudes of unitary events comprising Nav1.8-STTN EPSCs between recordings made in slices from SNI and sham mice. To this end, Nav1.8-STTN EPSCs were recorded under conditions in which extracellular Ca^2+^ was replaced by 1 mM Sr^2+^. This bivalent ion substitution resulted in the asynchronous release of synaptic vesicles from presynaptic terminals, revealing the asynchronous EPSCs (aEPSCs), which are the unitary events that constitute Nav1.8-STTN EPSCs evoked at normal calcium concentration (Oliet et al., 1996; Xu-Friedman and Regehr, 2000; Cheng et al., 2011). To analyze the mean aEPSC amplitude, a 500 ms window was placed immediately after the photostimulation, and the rise and decay phases of all EPSCs within the window that were not contaminated by other events (as marked with asterisks in Fig. 4*B*1) were selected and averaged (Fig. 4*B*2). For each L1-STTN, the events used for analysis were collected from all trials (at least 15) in the recording. We found that the mean amplitude of unitary aEPSCs did not differ; the mean amplitude averaged over six L1-STTNs from six SNI mice and five L1-STTNs from five shame mice was 21.3 ± 2.0 pA and 22.8 ± 2.4 pA, respectively (*p* = 0.64, unpaired *t* test; Fig. 4*B*3). Taken together, the results of the AMPAR/NMDAR ratio and aEPSCs confirm that there is no postsynaptic modulation of synaptic transmission by NRsNav1.8 on L1-STTNs after SNI.

### SNI increases a small portion of L1-STTNs exhibiting pERK-IR

**Figure 5.**
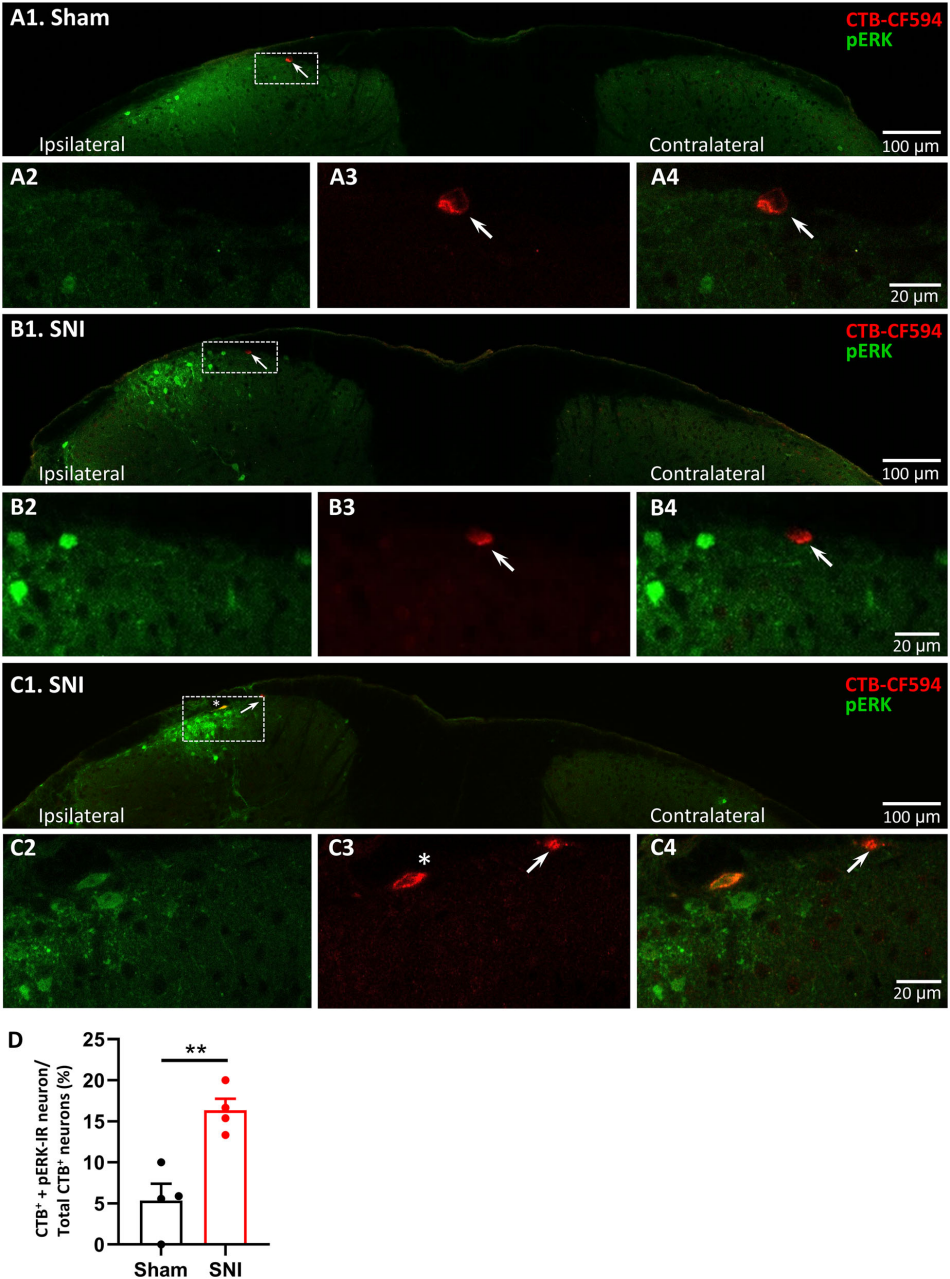
SNI results in only a small increase in the number of L1-STTNs showing pERK-IR in the lumbar segments. ***A***, Overlay of CTB-CF594 (red) and pERK-IR (green) signal in the DH at low magnification from a representative sham mouse experiment. The area enclosed by the dashed rectangle is highlighted and magnified; it shows that a pERK-IR neuron marked by the arrow (***A*2**) is not labeled by CTB-594 (***A*3**), as evidenced by no overlay of the two signals (***A*4**). ***B***, ***C***, Two representative experiments of SNI mice show the cases where L1-STTNs show either no pERK-IR (***B***) or pERK-IR (***C***) after SNI. The images shown are an overlay of CTB-CF594 (red) and pERK-IR (green) signals from the DH at low magnification (***B*1**, ***C*1**). When the areas enclosed by the dashed rectangles are highlighted and magnified, it can be seen that a pERK-IR neuron marked by the arrow (***B*2**) is not labeled with CTB-CF594 in one case (***B*3**), as shown by the overlay of the two signals (***B*4**). In the other case, the pERK-IR neuron marked with an arrow (***C*2**) is not labeled with CTB-CF594, whereas the pERK-IR neuron marked with an asterisk (***C*3**) is labeled with CTB-CF594, as shown by the overlay of the two signals (***C*4**). In the images, ipsilateral and contralateral refer to the DH ipsilateral and contralateral to the injured leg, respectively. ***D***, Pooled results from four SNI and four sham mice. Each symbol in the plots indicates the result from one mouse; bars indicate the means, and capped lines indicate the SEM. The asterisks indicate significant differences at the *p* < 0.01 level.

A biochemical hallmark of chronic pain is the NMDAR-dependent increase in pERK levels in the DH (Ji et al., 1999, 2009; Wei et al., 2006). At the synapses of nociceptive input from the lateral parabrachial nucleus (LPB) to the capsular central amygdaloid neurons, increased pERK levels upon nociceptive stimulation have been shown to not only serve as an indicator of neuronal activation but also to play a key role in the development of behavioral hypersensitivity and the underlying postsynaptic enhancement of synaptic transmission (Carrasquillo and Gereau, 2007; Cheng et al., 2011). Accordingly, the observation of no postsynaptic modulation in our electrophysiological experiments seems to contradict the results of these previous reports. A possible explanation could be that other neurons, but not the L1-STTN, in the DH of SNI mice show an increase in pERK levels. We tested this hypothesis by performing CTB-594 retrograde tracing combined with IHC using antibodies against pERK and comparing the staining profiles of L1-STTNs between sham and SNI mice. Again, Nav1.8^Chr2^ mice first received an infusion of CTB-594 into the VB thalamus to label L1-STTNs, followed by sham or SNI surgery 7 d after CTB-594 infusion. Thirty minutes but not 3 d after nerve transection for SNI or nerve exposure only for sham, mice were subjected to IHC and histochemical procedures. The rationale for not performing pERK staining 3 d after SNI, as in the rest of the experiments, is that this series aims to investigate whether the inducing but not maintaining role of ERK signaling (Ji et al., 1999, 2009; Zhuang et al., 2005; Wei et al., 2006), which may not be necessary and would have declined during the transitional period of pain chronification, is activated in L1-STTNs immediately after SNI. Consistent with previous studies (Ji et al., 1999; Wei et al., 2006), we found that there was a robust increase in pERK levels in the ipsilateral (but not contralateral) DH of SNI mice, while the sham mice showed only a small increase (Fig. 5). On average, the number of L1-STTNs in the lumbar segments 2–5 per mouse was 16.5 ± 1.6 and 14.3 ± 1.9 in sham mice (*n* = 4) and SNI mice (*n* = 4), respectively. In sham mice, only 5.4 ± 2.1% of the L1-STTNs showed pERK immunoreactivity (IR; Fig. 5*A,D*). In SNI mice, the robust increase in pERK-IR signal in the DH was associated with a small but significant increase in the percentage of L1-STTNs showing pERK-IR (16.3 ± 1.4%, *p* = 0.0045, unpaired *t* test; Fig. 5*B–D*). Taken together, the IHC results showing that <11% of the total L1-STTNs in the lumbar segments were activated, as indicated by the increased pERK level after SNI, supported the electrophysiological observations of a mild postsynaptic modulation of synaptic transmission from NR^Nav1.8^ to L1-STTNs after SNI.

### Spiking patterns of L1-STTNs before and after SNI

**Figure 6.**
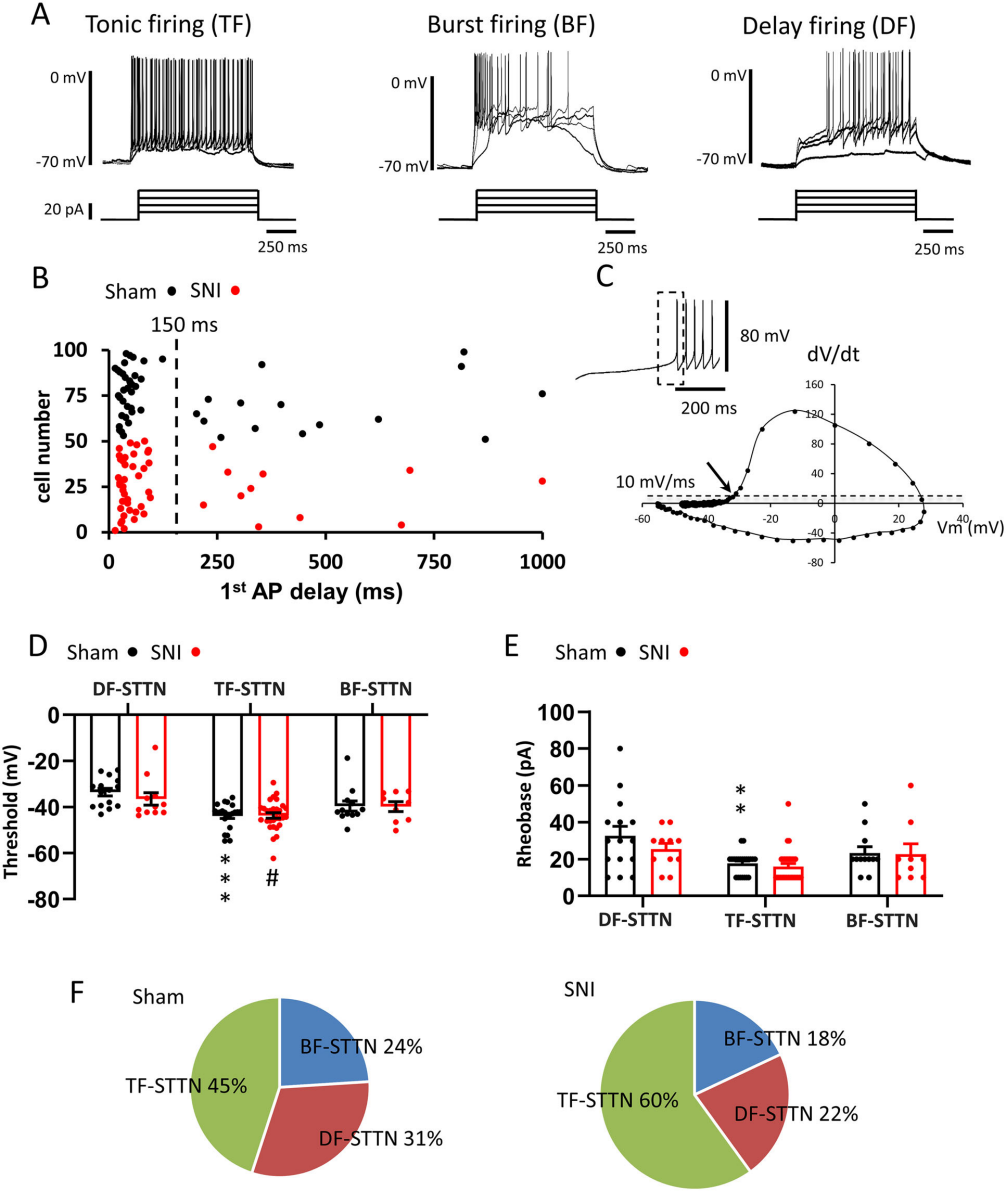
Spiking patterns of L1-STTNs in sham and SNI mice. ***A***, Representative recordings from L1-STTNs in sham mice show tonic firing (TF-STTN; left traces), burst firing (BF-STTN; middle traces), and delay firing (DF-STTN; right traces). The top traces show the responses of the recorded neurons to the current injection paradigms shown in the bottom traces. ***B***, The plot shows the distribution of L_1st-AP_ collected from 99 L1-STTNs. Data collected from SNI and sham mice are numbered from 1 to 50 (red symbols) and from 51 to 99 (black symbols), respectively. The dashed line shows the section of L1-STTNs with short L_1st-AP_ from those with long L_1st-AP_. ***C***, A representative example shows the AP threshold measurement of an L1-STTN. The top left plot shows the L1-STTN response to the current injection at rheobase intensity. The plot shows the differentiation of Vm as a function of time (dV/dt) of the first AP, marked by the dashed square of the plotted trace. The Vm at which dV/dt shows a sudden and rapid increase (marked with an arrow) is taken as the AP threshold. ***D***, ***E***, Plots show AP threshold (***D***) and rheobase (***E***) for each L1-STTN type collected from sham (black) and SNI (red) mice. Each symbol shows data collected from one L1-STTN from a sham or SNI mouse. Bars indicate the mean, and capped lines indicate the SEM. The asterisks denote significant differences compared with DF-STTN of sham mice at *p* < 0.01 (**) or <0.001 (***); # denotes significant differences compared with DF-STTN of SNI mice at *p* < 0.05. ***F***, Pie chart summarizing the percentages of DF-STTN, TF-STTN, and AF-STTN recorded from sham (top) or SNI (bottom) mice.

Previous studies have shown that superficial DH neurons exhibit distinct spiking patterns in response to depolarizing current injections at resting membrane potential (Ruscheweyh and Sandkühler, 2002; Ruscheweyh et al., 2004; Agashkov et al., 2019; Sinha et al., 2021). In these studies, the delay in AP generation, a feature that has been shown to involve activation of A-type voltage-gated K^+^ channels in DH neurons as well as in other neuronal types in the brain (Liss et al., 2001; Min et al., 2010), appears to be a key parameter for categorizing DH neuron types based on their firing properties. Interestingly, we found that although the neurons selected for whole-cell recording in this study were limited to those labeled with CTB-594, the recorded L1-STTNs still exhibited distinct spiking patterns in response to the injection of depolarizing current pulses (intensities, 0–80 pA; increment, 10 pA; duration, 1 s) with Vm held at ∼ −70 mV (Fig. 6*A*). In this series of experiments, a total of 99 L1-STTNs were sampled for analysis (49 L1-STTNs from 35 SNI mice and 50 L1-STTNs from 35 sham mice; Table 1). As shown in Figure 6*B* (dashed line), the delay in AP generation, which measures the latency from the onset of current injection to the peak of the first spike (L_1st-AP_), also appeared to be a good parameter for separating L1-STTNs recorded from sham mice into two groups. For the L1-STTNs categorized as no-delay and delayed firing, their L_1st-AP_ was 47.32 ± 2.69 and 470.53 ± 49.75 ms, respectively (*p* < 0.0001 Mann–Whitney *U* test). In some of the L1-STTNs without delay firing, they also displayed AP adaptation by showing cessation of APs before the end of the current injection (Fig. 6*A*), and such a feature was not observed in all L1-STTNs with delay firing. Accordingly, we categorized L1-STTNs into three types (Ruscheweyh et al., 2004; Agashkov et al., 2019): the delay firing (DF) STTNs, which have a long L_1st-AP_ and show no AP adaptation; the tonic firing (TF) STTNs, which have a short L_1st-AP_ and show no AP adaptation; and the burst firing (BF) STTNs, which have a short L_1st-AP_ and show AP adaptation. In sham mice, DF-STTNs, TF-STTNs, and BF-STTNs account for 31%, 45%, and 24% of the total (49) L1-STTNs, respectively (Table 1).

**Table 1.** Spiking properties of L1-STTNs

	L_1st-AP_ (ms)	AP threshold (mV)	Rh (pA)
Sham (*n* = 49)			
DF-STTN (*n* = 15)	490.51 ± 68.92^a^	−33.57 ± 1.63	32.67 ± 5.11
TF-STTN (*n* = 22)	43.75 ± 3.45	−43.87 ± 1.15^a^	17.73 ± 1.30^a^
BF-STTN (*n* = 12)	46.84 ± 8.49	−39.61 ± 2.23	23.33 ± 3.33
SNI (50)			
DF-STTN (*n* = 11)	443.28 ± 73.67^a^	−29.05 ± 7.51	25.45 ± 3.12
TF-STTN (*n* = 30)	53.56 ± 4.59	−43.68 ± 1.21^a^	16.00 ± 1.63
BF-STTN (*n* = 9)	35.91 ± 5.25	−39.84 ± 2.13	22.78 ± 5.60

a Denotes significant difference in L_1st-AP_, AP threshold, and Rh compared with the other two L1-STTN types within the same operation group (SNI or sham). For each type of L1-STTN, there are no significant differences in L_1st-AP_, AP threshold, and Rh between SNI and sham mice.

Interestingly, the criteria for L1-STTN grouping also applied well to the data collected from SNI mice. First, the L_1st-AP_ distribution pattern of SNI mice (Fig. 6*B*, red circle) is very similar to that of sham mice, with a cut of the L_1st-AP_ at 150 ms and spike adaptation also well separating the L1-STTNs of SNI mice into DF-STTNs, TF-STTNs, and BF-STTNs. Furthermore, when comparing the L_1st-AP_ of L1-STTNs recorded from sham and SNI mice, no significant difference was found for each type of L1-STTN (Table 1). Therefore, we next analyzed whether two key physiological properties, action potential threshold (Fig. 6*C*) and rheobase (Rh), defined as the minimum current intensity required to induce AP, exhibited differences among the three types of L1-STTNs in sham and SNI mice. The results show that the three types of L1-STTNs are significantly different in their AP thresholds in both sham and SNI mice; however, when compared between sham and SNI mice, no significant difference was found in all three types of L1-STTNs (Fig. 6*D*, Table 1). Specifically, a significant difference is found between DF-STTN and TF-STTN (*p* = 0.0002, two-way ANOVA followed by post hoc Tukey's multiple-comparisons test), but not between DF-STTN and BF-STTN (*p* = 0.19), nor between TF-STTN and BF-STTN in sham mice (*p* = 0.49; Fig. 6*D*, black circle). Similarly, a significant difference is found between DF-STTN and TF-STTN (*p* = 0.0393), but not between DF-STTN and BF-STTN (*p* = 0.88), nor between TF-STTN and BF-STTN in SNI mice (*p* = 0.66; Fig. 6*D*, red circle). No significant difference in AP threshold was found between sham and SNI mice for each L1-STTN (Fig. 6*D* and Table 1). Consistent with the analysis of AP threshold, differences in Rh were also observed among the three L1-STTN types in both sham and SNI mice (Fig. 6*E*). In sham mice, a significant difference in Rh is found between DF-STTN and TF-STTN (*p* = 0.0045), but not between DF-STTN and BF-STTN (*p* = 0.35), nor between TF-STTN and BF-STTN (*p* = 0.78; Fig. 6*E*, black circle). However, there was no difference found between DF-STTN and TF-STTN (*p* = 0.232), between DF-STTN and BF-STTN (*p* = 0.99), or between TF-STTN and BF-STTN in SNI mice (*p* = 0.67; Fig. 6*E*, red circle, and Table 1). For each L1-STTN type, Rh also shows no significant difference between sham and SNI mice (Fig. 6*E* and Table 1). Therefore, it appears that SNI switched a small population of DF-STTN and BF-STTN patterns to the TF-STTN pattern, as there was a small increase in the percentage of TF-STTNs (approximately 15%) and the corresponding small decrease in the percentages of DF-STTNs and BF-STTNs after SNI (Fig. 6*F* and Table 1). However, the difference in cell type proportions is not significant by the *χ*^2^ test (*p* = 0.32).

## Discussion

Using retrograde tracing combined with optogenetics, we were able to record EPSCs from DRG neurons expressing Nav1.8, most of which are peptidergic or nonpeptidergic nociceptors (collectively referred to in this study as NR^Nav1.8^; Akopian et al., 1996), onto L1-STTNs. We found that peripheral nerve injury could induce an increase in the strength of this synapse type and that the effect was due to presynaptic but minor postsynaptic modulation (if any). The minor postsynaptic modulation could be attributed to the fact that SNI resulted in only a small increase in the number of L1-STTNs (approximately 10% compared with sham surgery; Fig. 5*D*), which exhibited the elevated levels of pERK, a key mediator of synaptic plasticity involving an increase in the number/function of receptors at glutamatergic synapses (Zhu et al., 2002; Qin et al., 2005; Cheng et al., 2011). Compared to sham surgery, we also found a small increase (by 15%; Fig. 6*F*) in the number of TF-STTNs, which have the lowest AP threshold and rheobase among the three L1-STTN types after SNI. Interestingly, the increase in the percentage of TF-STTNs was compatible with the increase in the number of L1-STTNs showing pERK-ir after SNI. Taken together, the results of this study demonstrate that peripheral nerve injury induces a presynaptic modulation of synaptic transmission from peptidergic/nonpeptidergic nociceptors to L1-STTNs, whose axons constitute the lateral spinothalamic tract.

Our argument for a presynaptic enhancement of synaptic transmission from NR^Nav1.8^ to L1-STTNs is based on the results of FR, PPR, and sEPSCs analyses. We found that the enhancement of Nav1.8-STTN EPSCs after SNI was associated with a significant decrease in FR and PPR and an increase in frequency but not in the amplitude of sEPSCs, suggesting that peripheral nerve injury may result in enhanced glutamate release from nociceptive terminals in the DH. In contrast to the FR and PPR analysis, in which the optogenetic method provides specificity for stimulation of NR^Nav1.8^ axonal terminals, the analysis of sEPSCs lacks such specificity because the events analyzed include all synapses on the L1-STTNs. Despite this weakness, the results of the sEPSCs analysis are consistent with the observations of the FR and PPR studies. Meanwhile, a striking morphological feature of the L1-STTNs described in this study is that dendritic arborization was restricted to the L1 of the DH, where the Cre-dependent eYFP signal, namely, NR^Nav1.8^ axonal terminals, was distributed. This observation suggests that the majority of synaptic inputs to L1-STTNs originate from NRNav1.8 axonal terminals, making the analysis of sEPSCs valuable. The present finding of presynaptic enhancement of glutamate release from NR^Nav1.8^ terminals is not only consistent with the findings of Daou et al. (2016), who reported that long-term optogenetic silencing of NR^Nav1.8^ in anesthetized Nav1. 8^Arch^ mice, in which the inhibitory archaerhodopsin-3 (Arch) proton pump was delivered to NR^Nav1.8^ using the same Nav1.8^Cre^ mouse line as in the present study, resulted in poststimulation analgesia with a significant reduction in mechanical and thermal hypersensitivity under inflammatory and neuropathic conditions. A possible candidate molecular substrate for mediating presynaptic enhancement after SNI could be the presynaptic NMDARs in the DH of the spinal cord (for review, see Deng et al., 2019). Previous studies have shown the presence of an immunoreactivity signal of the GluN1 subunit of NMDAR at the terminals of both unmyelinated and myelinated primary afferent nerves in the superficial DH (H. Liu et al., 1994; Lu et al., 2003). Furthermore, ex vivo electrophysiological recordings from neurons in L1/L2 of the DH have confirmed these morphological observations (Zeng et al., 2006; Zhao et al., 2012; Yan et al., 2013; L. Li et al., 2016). Taken together, these results suggest that constitutive activation of presynaptic NMDARs in DH neurons could potentiate glutamate release from nociceptive primary afferent terminals in various neuropathic pain models and are consistent with the main finding of this study regarding the presynaptic enhancement of synaptic transmission of NR^Nav1.8^ to L1-STTNs after peripheral nerve injury.

The results of this study are insufficient to determine whether it was the central terminals of the injured and/or spared DRG neurons in the DH that developed presynaptic modulation after SNI; namely, it is uncertain whether it was the injured and/or spared DRG neurons that would have upregulated the expression of presynaptic NMDARs (if these molecules indeed mediate presynaptic enhancement) at their central terminals. A recent study by Yeh et al. (2021) using an intravital imaging method for longitudinal observation of peripheral nerves expressing Nav1.8 reported that in the toe tips of mice receiving SNI surgery, degeneration of free nerve endings in the epidermis and underlying nerve plexuses could occur within 2 and 3 d, respectively, after nerve injury. This study also shows that some of the Nav1.8-expressing nerve plexuses and free nerve endings in the fifth toe survived and mostly sprouted between 7 and 28 d. Because the timing of degeneration and nerve sprouting in the fifth toe parallels the initiation and maintenance phases of behavioral hypersensitivity induced by mechanical stimulation, the authors argue that both injured and intact nerve fibers are involved in neuropathic pain (Yeh et al., 2021); specifically, nerve degeneration is related to the initiation of evoked pain, and nerve sprouting is related to the maintenance of evoked pain. However, we did not observe any significant changes in the density and distribution profile of the central ends of NR^Nav1.8^ axonal terminals after SNI. Accordingly, it is unlikely that the central ends of NR^Nav1.8^ axonal terminals also underwent degeneration in the injured and sprouting in the spared, as suggested by Yeh et al. (2021) at their peripheral ends. Because we prepared the ex vivo spinal cord slices for electrophysiological recording and post hoc histochemistry 3 d after nerve injury, the sprouting of the spared NR^Nav1.8^ axonal terminals at the peripheral site as suggested by Yeh et al. (2021) may not have occurred yet. Nevertheless, it should be reemphasized that the present results are not sufficient to exclude the possibility of upregulation of the molecular substrate mediating presynaptic enhancement at central terminals of spared NR^Nav1.8^ after SNI.

In contrast to presynaptic enhancement, we argue that there is little (if any) postsynaptic modulation of synaptic transmission from NR^Nav1.8^ to L1-STTNs after peripheral nerve injury, based on the observation that there was no significant change after SNI in two parameters commonly used to indicate postsynaptic modulation (AMPAR/NMDAR ratio and amplitude of aEPSCs). These observations and the arguments derived from them seem to be controversial in many previous studies that reported changes in surface AMPAR/NMDAR expression in superficial DH neurons in animals with peripheral injury or inflammatory pain (Guo and Huang, 2001; Nagy et al., 2004; Katano et al., 2008; Vikman et al., 2008; Park et al., 2009; Kopach et al., 2011; S. R. Chen et al., 2013; Sullivan et al., 2017; J. Li and Baccei, 2019; for review, see Youn et al., 2013). However, since ERK-related pathways have been shown to play a key role in regulating AMPAR trafficking for the expression of NMDAR-dependent LTP (Zhu et al., 2002; Qin et al., 2005), the results of the present IHC studies showing a small but significant increase in the number of pERK-ir DH neurons after SNI are consistent with these previous studies. Importantly, L1-STTNs accounted for a small proportion (approximately only 5%) of pERK-ir neurons in the DH of sham mice, and the number increased by only approximately 10% after SNI. It should be noted that the percentages of L1-STTNs exhibiting pERK-ir were obtained based on the number of neurons summed from all lumbar segments and averaged over six mice. Given that approximately two L1-STTNs were observed in L1 of a spinal cord slice, the L1-STTN recorded in each experiment should either have elevated pERK levels or not, with the possibility that the former would be much lower than the latter. Accordingly, a possible explanation for the discrepancy in electrophysiological findings between the present and previous studies could be that, compared with the previous studies, the whole-cell recordings were specifically made from L1-STTNs and the NR^Nav1.8^ were selectively stimulated in this study. In fact, we validated and recorded monosynaptic Nav1.8-STTN EPSCs, which is important for exploring pain mechanisms at the DH level, because there are different types of neurons in the superficial DH of the spinal cord, including GABAergic interneurons (Al-Khater et al., 2008; Zholudeva et al., 2021). We believe that the majority of L1-STTNs recorded from SNI slices did not have elevated pERK (postsynaptic modulation) levels, explaining the results of no significant difference in AMPAR/NMDAR ratio and unitary aEPSC amplitude compared with data from sham slices. It is very likely that some DH neurons with elevated pERK levels, indicating postsynaptic modulation of their excitability after SNI, were actually GABAergic interneurons that play a role in modulating the strength of nociceptive input to L1-STTNs through their axon–axon connections with the terminals of NR^Nav1.8^ (Eccles et al., 1963; Fernandes et al., 2020). A recent study by Rankin et al. (2024) shows that SNI disrupts temporal processing in the DH by altering GABAergic interneurons that express parvalbumin. Interestingly, although not analyzed further, we observed an increase in asynchronous glutamate release from NR^Nav1.8^ after nerve injury in this study (Fig. 3*A*, asterisk). Taken together, based on the present and previous results, we suggest that peripheral nerve injury could cause a presynaptic enhancement of glutamate release from the central end of nociceptor terminals in the DH, which could readily trigger postsynaptic modulation of numerous DH neurons, as indicated by the elevated pERK-ir levels, except for the L1-STTN, which may have a very high threshold for postsynaptic modulation. The mechanism underlying the very high threshold for postsynaptic modulation and the physiological significance of this feature of L1-STTNs require further investigation.

When examining possible changes in the firing pattern of L1-STTNs before and after SNI, we also observed that the percentage increases in TF-STTNs and L1-STTNs showing pERK-ir by SNI were very similar. Since TF-STTNs have the lowest AP threshold and rheobase among the three L1-STTN types, this result shows that SNI may promote the excitability of a small population of L1-STTNs. Based on this finding, we speculate that a few DF-STTNs and BF-STTNs may switch their firing properties to that of TF-STTNs after SNI. A potential molecular player in the SNI-induced firing pattern switch could be the voltage-gated K^+^ channel subunit Kv4.2, which mediates A-type K^+^ currents (I_A_). The Kv4.2-mediated I_A_ exhibits gating properties of rapid activation at subthreshold membrane voltage and rapid inactivation (McCormick and Huguenard, 1992), which could provide a transient voltage barrier to AP generation. Accordingly, neurons with a large amount of I_A_ typically show a delay in the generation of the first AP when they receive a depolarizing current injection with an intensity greater than their rheobase (Liss et al., 2001; Min et al., 2010). Interestingly, accumulating evidence has shown that the functionality of Kv4.2 channels is downregulated upon phosphorylation by ERK in many brain neurons (Yang et al., 2001; Yuan et al., 2002) and DH neurons (Hu et al., 2003, 2006; Hu and Gereau, 2003). Furthermore, in DH neurons, modulation of Kv4.2 by ERK signaling pathways has been shown to contribute to nociceptive plasticity and central sensitization associated with chronic inflammatory pain conditions (Hu et al., 2006, 2007). Therefore, it is likely that although the threshold is very high, when postsynaptic modulation is triggered in an L1-STTN, as indicated by the elevated pERK signal subsequently mediating a downregulated Kv4.2 channel, the activated L1-STTN would exhibit a tonic firing pattern and higher excitability.

In conclusion, the present results demonstrate that while peripheral nerve injury generally induces presynaptic enhancement of glutamate release from NR^Nav1.8^, it also causes postsynaptic modulation of DH neurons, with the exception of L1-STTNs. Because L1-STTNs play a role in transmitting nociceptive information to the VPL thalamus and then to S1 with discriminative functions, and other DH neurons could modify the outputs of L1-STTNs, such synaptic organizations and plasticity properties may be able to ensure reliable pain signaling to SI for the discriminative functions, as well as allow adaptive modulation of pain signaling to brain regions for other aspects of pain signal processing, such as the aversive and emotional components of pain.

## Synthesis

Reviewing Editor: Jennifer Dulin, Texas A&M University Decisions are customarily a result of the Reviewing Editor and the peer reviewers coming together and discussing their recommendations until a consensus is reached. When revisions are invited, a fact-based synthesis statement explaining their decision and outlining what is needed to prepare a revision will be listed below. The following reviewer(s) agreed to reveal their identity: NONE.

We found that the authors were very responsive to the previous reviewer's comments. Only very minor corrections are requested at this stage.

Reviewer's comments:

The reviewer is satisfied by the authors answers and modifications incorporated to the manuscript.

Last concern of the reviewer: there is a growing body of studies in the pain field pointing at sex differences in pain. It is unclear from the animal section whether the authors used males or females or both. A clue seems to be found in the surgery section of the material and methods that only males were used. The authors need to clarify sex and age of the mice used in the study, and if no female mice were involved, add a justification for that choice. If females were involved, the authors need to mention whether sex differences were observed or not, a table would be then helpful males vs females.

## Author Response

Last concern of the reviewer:

There is a growing body of studies in the pain field pointing at sex differences in pain. It is unclear from the animal section whether the authors used males or females or both. A clue seems to be found in the surgery section of the material and methods that only males were used. The authors need to clarify sex and age of the mice used in the study, and if no female mice were involved, add a justification for that choice. If females were involved, the authors need to mention whether sex differences were observed or not, a table would be then helpful males vs females.

Response:

Thank you very much! As requested, we have clarified the sex of the mice used for all experiments (see red text on page 7, lines 3-5 and line 16). The justification for the choice is also provided (see blue text on page 7, lines 5 to 9). It states: This choice of animal sex is intended to avoid the potential effects of estrous cycle-related hormones at this stage. However, as a growing number of studies in the field of pain indicate sex differences in pain, this issue should be systematically investigated as a separate study in the future.
